# Relationships of serum soluble E-selectin concentration with insulin sensitivity and metabolic flexibility in lean and obese women

**DOI:** 10.1007/s12020-013-0025-9

**Published:** 2013-08-10

**Authors:** Agnieszka Adamska, Monika Karczewska-Kupczewska, Agnieszka Nikołajuk, Elżbieta Otziomek, Maria Górska, Irina Kowalska, Marek Strączkowski

**Affiliations:** 1 Department of Endocrinology, Diabetology and Internal Medicine, Medical University of Bialystok, M.C. Sklodowskiej 24a, 15-276 Bialystok, Poland; 2 Department of Prophylaxis of Metabolic Diseases, Institute of Animal Reproduction and Food Research, Polish Academy of Sciences, Olsztyn, Poland

**Keywords:** Markers of endothelial dysfunction, The metabolic syndrome, Substrate metabolism

## Abstract

The markers of endothelial dysfunction, including soluble E-selectin (sE-selectin), are related to insulin resistance, which is associated with metabolic inflexibility, i.e., impaired stimulation of carbohydrate oxidation and impaired inhibition of lipid oxidation by insulin. Endothelial dysfunction may also be important in the metabolic syndrome. The aim of our study was to analyze the association of sE-selectin with insulin sensitivity and metabolic flexibility in lean and obese women. We examined 22 lean women (BMI < 25 kg m^−2^) and 26 overweight or obese women (BMI > 25 kg m^−2^) with normal glucose tolerance. A hyperinsulinemic euglycemic clamp and indirect calorimetry were performed. An increase in the respiratory exchange ratio in response to insulin was used as a measure of metabolic flexibility. Obese women had lower insulin sensitivity (*P* < 0.01), higher plasma sE-selectin (*P* = 0.007), and higher the metabolic syndrome total *Z*-score (MS *Z*-score) (*P* < 0.0001). Insulin sensitivity was negatively correlated with sE-selectin level (*r* = −0.24, *P* = 0.04). sE-selectin was associated with the rate of carbohydrate oxidation at the baseline state (*r* = 0.31, *P* = 0.007) and was negatively correlated with metabolic flexibility (*r* = −0.34, *P* = 0.003). MS *Z*-score correlated positively with sE-selectin level and negatively with metabolic flexibility and insulin sensitivity (*r* = 0.49, *P* < 0.0001, *r* = −0.29, *P* = 0.04, *r* = −0.51, *P* < 0.0001, respectively). In multiple regression analysis we observed that the relationship between metabolic flexibility and sE-selectin (*β* = −0.36; *P* = 0.004) was independent of the other evaluated factors. Our data suggest that endothelial dysfunction as assessed by plasma sE-selectin is associated with metabolic flexibility, inversely and independently of the other estimated factors.

## Introduction

The National Cholesterol Education Program, Adult Treatment Panel III established the criteria of the metabolic syndrome (MS) (≥3 criteria: central obesity waist ≥88 cm in women or ≥102 cm in men; HDL <50 mg dL^−1^ in women; triglycerides ≥194 mg dL^−1^; hypertension ≥135/85 mmHg; fasting plasma glucose ≥110 mg dL^−1^) [1]. It is widely accepted that low levels of physical activity, as well as an over-abundance of food, are the main reasons for the metabolic syndrome where metabolic inflexibility (MI) is significant. MI is an impaired stimulation of carbohydrate oxidation and impaired inhibition of lipid oxidation in response to insulin [2]. The incidence and severity of MS is inversely related to fitness, and it is a marker of a sedentary lifestyle [3].

One of the methods used to show the severity of MS is the metabolic syndrome *Z*-score (MS *Z*-score), which can be assessed from the standardized individual component, i.e., waist circumference, blood pressure, lipid profile, glucose level, and physical activity. A lower score means a better, and a higher score indicates a worse, profile of individuals [3].

It seems that the role of endothelial dysfunction is important in MS [4, 5]. Endothelial dysfunction is related to the insulin resistance state. It could be an early stage in the atherosclerosis process, and leads to many diseases, such as cardiovascular events [6] and type 2 diabetes [7]. It can be detected by the measurement of plasma levels of soluble E-selectin (sE-selectin) and the soluble inter-cellular adhesion molecule-1 (sICAM-1) [8, 9], and is often found in overweight/obese subjects [6, 7, 9–14].

E-selectin is an endothelial cell-specific membrane glycoprotein which is required for the slow rolling of leukocytes on the endothelium during inflammation [15]. Many data have shown that sE-selectin reflects a low-grade chronic inflammation of the endothelium and independently predicts the risk of MS, cardiovascular disease, type 2 diabetes, and insulin resistance [5]. sE-selectin is also elevated in women with polycystic ovary syndrome [16], and also correlates with the levels of glucose, triglycerides, and uric acid [16].

As mentioned above, MS is a constellation of metabolic disturbances with carbohydrate and lipid metabolism fluctuation [17]. One of the hypothesis emphasizes that MS could be associated with endothelial dysfunction induced by inflammation and decreased mitochondrial content, where oxidative stress is one key component of this [18]. Insulin resistance is an important pathogenic factor of MS, and is especially connected with a low-grade chronic inflammation [19, 20]. Many data have highlighted a self-stimulating inflammatory cycle in metabolic inflexibility state [4]. This cycle starts with ectopic fat depots in the skeletal muscle, the liver, or the pancreas. These depots may be the source of oxidative stress [21, 22]. Moreover, some data have demonstrated that it could lead to suppressing mitochondrial function, especially the capacity for fuel usage [23]. The role of sE-selectin in this cycle has not been clear until now, especially its impact on metabolic flexibility.

Another marker of endothelial dysfunction is sICAM-1, which is elevated in patients with cardiovascular disease, and in young healthy adult offspring of parents with type 2 diabetes [24]. The markers of endothelial dysfunction, including soluble sE-selectin and sICAM-1 [25], may represent a link between obesity, insulin resistance, and related co-morbidities [6, 7, 9–13].

Other mediators, namely adiponectin, could have a protective role in endothelial dysfunction [26], and may exert an anti-atherogenic effect by vasodilatation, also independently of insulin sensitivity [6, 7, 9–13]. Adiponectin has been shown to have anti-atherogenic properties by suppressing the secretion of sE-selectin [13, 27]. Apart from the influence of adiponectin on endothelial function, this adipocytokine could have multiple effects on glucose and lipid metabolism [28–32]. It is not yet established whether this protein is more strongly associated with oxidative or non-oxidative glucose metabolism [29, 33, 34], or especially with lipid metabolism [35].

The aim of the present study was to analyze the association between plasma sE-selectin and whole-body insulin sensitivity, lipid and carbohydrate oxidation, and metabolic flexibility in lean and obese women.

## Subjects and methods

### Subjects

We examined 22 lean women (BMI < 25 kg m^−2^) and 26 overweight or obese women (BMI > 25 kg m^−2^) with a sedentary lifestyle. Obese/overweight subjects were recruited from the outpatient clinic of the Department of Endocrinology, Diabetology and Internal Medicine, Medical University of Bialystok, and from the medical staff and students. Control subjects were recruited from the medical staff and students. None of the participants had morbid obesity, cardiovascular disease, hypertension, infection, clinical or biochemical manifestation of hyperandrogenemia, or other serious medical problems. None of the participants took anti-inflammatory drugs (within the previous 3 months) or drugs known to affect glucose or lipid metabolism. All the subjects had regular menses. The studied women were non-smokers. Prior to entering the study physical examinations were performed. All the subjects underwent an oral glucose tolerance test (OGTT) and had normal glucose tolerance according to WHO criteria. All the subjects gave written informed consent prior to entering the study. The study protocol was approved by the Ethics Committee of the Medical University of Bialystok, Poland. It is worth mentioning that 71 % of the women had co-participated in our previous study [30, 36].

### Anthropometric measurements

BMI was calculated as body weight in kilograms divided by height in meters squared (kg m^−2^). Waist circumference was measured at the smallest circumference between the rib cage and the iliac crest, with the subject in the standing position. The percentage of body fat was estimated by bioelectric impedance analysis using the Tanita TBF-511 Body Fat Analyzer (Tanita Corp., Tokyo, Japan).

### Insulin sensitivity

Insulin sensitivity was evaluated by the euglycemic hyperinsulinemic clamp technique as described by DeFronzo et al. [37]. We recruited healthy, regularly menstruating women and the studies were performed 3–5 days after a spontaneous menses. Insulin (Actrapid HM, Novo Nordisk, Copenhagen, Denmark) was administered as a primed-continuous intravenous infusion for 2 h at 40 mU m^−2^ min^−1^, resulting in a constant hyperinsulinemia of approximately 75 mIU L^−1^. Arterialized blood glucose was obtained every 5 min, and a 20 % dextrose (1.11 mol l^−1^) infusion was administrated. The clamp was performed at 5 mmol l^−1^ glucose concentration. The glucose infusion rate approached stable values during the final 40 min of the study. The rate of the whole-body glucose uptake (*M* value) was calculated as the mean glucose infusion rate from 80 to 120 min, corrected for glucose space and normalized per kilogram of fat-free mass (*M*_ffm_).

### Lipid and carbohydrate oxidation

Whole-body lipid and carbohydrate oxidation rates were measured by indirect calorimetry using the ventilated hood technique (Oxycon Pro, Viasys Healthcare GmbH—Erich Jaeger, Hochberg, Germany) in order to calculate substrate oxidation from respiratory gas exchange (oxygen consumption and carbon dioxide production). The device was calibrated before each test using reference gases. The measurements were performed when the subjects were lying in a supine position during 30 min at the baseline (in the fasting state) and during the last 30 min of the clamp study. Each study was performed in a thermoneutral environment and was followed by 15-min relaxation. Non-oxidative glucose metabolism (NOGM) was calculated by subtracting the carbohydrate oxidation rate during hyperinsulinemia from the whole-body glucose disposal rate. The increase in respiratory exchange ratio (delta RER) in response to insulin was used as a measure of metabolic flexibility.

### Biochemical analyses

Fasting blood samples were collected from the antecubital vein prior to the clamp for the determination of plasma lipids, and sE-selectin, sICAM-1, and adiponectin levels. The samples were frozen at −70 °C until the analyses. Plasma glucose was measured immediately by the enzymatic method using a glucose analyzer (YSI 2300 STAT PLUS). Plasma insulin was measured with the monoclonal immunoradiometric assay (IRMA, Medgenix Diagnostics, Fleunes, Belgium). The minimum detectable concentration was 1 μIU ml^−1^, and the intra-assay and inter-assay coefficients of variation (CVs) were below 2.2 and 6.5 %, respectively. In this method human and animal proinsulins present no cross-reaction. Serum total and HDL-cholesterol and triglycerides (TG) were assessed by enzymatic methods using commercial kits produced by ANALCO-GBG, Poland. sICAM-1 and sE-selectin were measured with EASIA kits (R&D Systems Inc., USA). The minimum detectable concentration was 0.049 ng ml^−1^ for sICAM and 0.003 ng ml^−1^ for sE-selectin. The intra-assay and inter-assay CVs for sE-selectin were below 6.9 and 8.6 %, respectively, and for sICAM—below 5 and 6.8 %, respectively. Plasma adiponectin was measured with an RIA Kit (Linco Research, Inc. St. Charles, Missouri, USA) with a detection limit of 1 ng ml^−1^, and with intra-assay and inter-assay CVs below 6.3 and 9.5 %, respectively.

### The metabolic syndrome *Z*-score (MS *Z*-score)

One of the methods to show the severity of the metabolic syndrome is the metabolic syndrome *Z*-score. We calculated MS *Z*-score as the sum of individual component of MS (waist circumference, blood pressure, HDL-cholesterol, triglycerides, and fasting plasma glucose) after normalization of the variables for their standard deviation. The standardized HDL value was multiplied by −1 [3].

### Statistical analysis

**Fig. 1:**
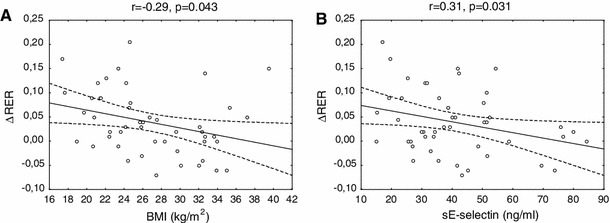
Relationship between BMI and metabolic flexibility (**a**) and sE-selectin and metabolic flexibility (**b**) in the entire studied group (*n* = 48)

The statistic analysis was performed with the Statistica 10.0 program (StatSoft, Krakow, Poland). The differences between the groups were evaluated with an unpaired Student’s *t* test. The variables with abnormal distribution (TG, adiponectin, sE-selectin, and substrate oxidation) were presented as medians (interquartile ranges). Therefore, we used the non-parametric Mann–Whitney test for these values. The relationships between the variables were estimated with simple and multiple regression analysis. We also designed a model of stepwise regression analysis with metabolic flexibility as a dependent variable. In model 1 BMI and age were included as predictors. In model 2 insulin sensitivity was also entered, whereas in model 3 sE-selectin was also added. Finally, in model 4, age, BMI, waist girth, fasting glucose, insulin, TG, adiponectin, sE-selectin, and insulin sensitivity were studied as independent variables. The level of significance was accepted at *P* value less than 0.05 (Fig. 1).

## Results

The clinical characteristics of the studied groups are shown in Table 1. Obese subjects had lower insulin sensitivity (*P* = 0.03), and higher triglycerides and fasting insulin levels (*P* = 0.009; *P* = 0.01, respectively). Plasma soluble E-selectin was higher in the obese group (*P* = 0.007), whereas sICAM-1 and adiponectin levels did not differ between the two groups (Table 1).

**Table 1:** Clinical and biochemical characteristics of the studied groups

	Lean subjects (*n* = 22)	Obese subjects (*n* = 26)
Age (years)	24.3 ± 5.8	25.1 ± 5.6
Body weight (kg)	60.7 ± 7.2	85.8 ± 12.7*
BMI (kg m^−2^)	22.0 ± 2.2	31.0 ± 3.8*
Waist girth (cm)	72.3 ± 6.0	95.6 ± 11.9*
FFM (kg)	46.1 ± 5.2	51.2 ± 7.1*
Body fat (%)	24.1 ± 7.1	39.4 ± 8.2*
Total cholesterol (mg dl^−1^)	182.0 ± 37.5	168.9 ± 32.7
Serum TG (mg dl^−1^)	59.0 (48–74)	93.8 (58–115)*
Fasting glucose (mg dl^−1^)	82.9 ± 7.3	81.8 ± 7.5
Fasting insulin (μIU ml^−1^)	10.4 ± 4.4	15.7 ± 4.4*
Serum adiponectin (μg ml^−1^)	13.1 (11.4–21.3)	11.5 (8.2–13.9)
sICAM-1 (ng ml^−1^)	254.3 ± 42.6	289.8 ± 89.9
sE-selectin (ng ml^−1^)	31.4 (25.4–40.9)	42.7 (33.8–54.4)*
*M* (mg kg_ffm_^−1^ min^−1^)	10.9 ± 3.7	8.8 ± 2.9*
MS total *Z*-score	−2.1 ± 2.6	1.8 ± 1.9*

Data are presented as mean ± SD and non-normally distributed variables are showed as medians (interquartile range)

* *P* < 0.05 in obese vs. lean subjects

*BMI* body mass index, *TG* triglycerides, *sICAM* soluble inter-cellular adhesion molecule-1, *sE-selectin* soluble E-selectin, *FFM* fat-free mass, *M* whole-body glucose uptake normalized per kg of fat-free mass, *MS* total *Z*-score-the metabolic syndrome total *Z*-score

Fasting rate glucose oxidation did not differ between the studied groups, whereas the rate of lipid oxidation was lower in obese subjects (*P* = 0.46; *P* = 0.03). During the clamp RER and the rate of carbohydrate oxidation were lower (*P* = 0.0005; *P* = 0.001, respectively), whereas lipid oxidation was higher in the obese subjects (*P* = 0.0003). Metabolic flexibility was lower in the obese group (*P* = 0.002) (Table 2), whereas delta carbohydrate oxidation (change in carbohydrate oxidation in response to insulin) was higher in the lean subjects, and delta lipid oxidation (change in lipid oxidation in response to insulin) was lower in this group (*P* = 0.0006, *P* = 0.02, respectively).

**Table 2:** Respiratory exchange ratio, nutrient oxidation rate, NOGM, and metabolic flexibility before and during clamp in lean and obese subjects

	Lean subjects (*n* = 22)	Obese subjects (*n* = 26)
RER-basal	0.80 ± 0.04	0.80 ± 0.06
RER-clamp	0.88 ± 0.07	0.81 ± 0.05*
COx-basal (mg kg_ffm_^−1^ min^−1^)	1.19 (0.73–1.72)	1.23 (0.83–2.23)
LOx- basal (mg kg_ffm_^−1^ min^−1^)	0.93 (0.71–1.26)	1.23 (0.99–1.55)*
COx-clamp (mg kg_ffm_^−1^ min^−1^)	2.64 (2.10–2.98)	1.42 (1.09–1.93)*
LOx-clamp (mg kg_ffm_^−1^ min^−1^)	0.44 (0.28–0.82)	1.19 (0.87–1.39)*
NOGM (mg kg_ffm_^−1^ min^−1^)	8.42 ± 2.92	7.16 ± 3.59
Delta RER	0.08 ± 0.08	0.01 ± 0.06*

Data are presented as mean ± SD and non-normally distributed variables are showed as medians (interquartile range)

* *P* < 0.05 in obese vs lean subjects

*RER-basal* respiratory exchange ratio before clamp, *RER-clamp* respiratory exchange ratio during clamp, *COx-basal* rate of carbohydrate oxidation in the basal state, *LOx-basal* rate of lipid oxidation in the basal state, *COx-clamp* rate of carbohydrate oxidation during hyperinsulinemia, *LOx-clamp* rate of lipid oxidation during hyperinsulinemia, *NOGM* non-oxidative glucose metabolism, *delta RER* change in respiratory exchange ratio in response to hyperinsulinemia

Insulin sensitivity was positively correlated with the plasma adiponectin level and negatively to the sE-selectin level (*r* = 0.38, *P* = 0.007, *r* = −0.29, *P* = 0.03, respectively). sE-selectin correlated positively with BMI (*r* = 0.40, *P* = 0.004), waist circumference (*r* = 0.43, *P* = 0.002), percentage of body fat (*r* = 0.43, *P* = 0.003), and plasma triglycerides level (*r* = 0.40, *P* = 0.004).

We found that sE-selectin was negatively correlated with metabolic flexibility (*r* = −0.31, *P* = 0.031), NOGM (*r* = −0.37, *P* = 0.008), and the increase in carbohydrate oxidation (*r* = −0.29, *P* = 0.04) in response to hyperinsulinemia.

Adiponectin was associated with RER (*r* = 0.34, *P* = 0.01) and the rate of glucose oxidation (*r* = 0.36, *P* = 0.01) during the clamp.

We did not find a statistically significant correlation between sICAM and insulin sensitivity or fuel metabolism (all *P* > 0.05).

The metabolic syndrome total *Z*-score was lower in the lean subjects in comparison to the obese (*P* < 0.0001) (Table 1). MS *Z*-score correlated positively with sE-selectin level and negatively with metabolic flexibility and insulin sensitivity (*r* = 0.49, *P* < 0.0001, *r* = −0.29, *P* = 0.04, *r* = −0.51, *P* < 0.0001, respectively).

In multiple regression analysis we observed that the relationship between metabolic flexibility and sE-selectin (*β* = −0.34; *P* = 0.04) was independent of age, BMI, waist circumference, fasting glucose and insulin level, insulin sensitivity, triglycerides, and adiponectin. In model 1 of the stepwise regression analysis BMI (*R* ^2^ = 0.09, *P* = 0.04) and age (*R* ^2^ = 0.04) together explained 13 % of the variance in metabolic flexibility. Model 2 gave an identical result, as insulin sensitivity did not enter the regression model. In model 3 sE-selectin (*R* ^2^ = 0.11, *P* = 0.018), together with age (*R* ^2^ = 0.04) and BMI (*R* ^2^ = 0.03), explained 18 % of metabolic flexibility variation. In model 4 sE-selectin (*R* ^2^ = 0.11, *P* = 0.018), fasting insulin (*R* ^2^ = 0.06), age (*R* ^2^ = 0.04), adiponectin (*R* ^2^ = 0.03), and insulin sensitivity (R^2^ = 0.02) explained 26 % of metabolic flexibility variation.

## Discussion

Our results demonstrated for the first time that glucose and lipid metabolism is associated with sE-selectin, and that this adhesion molecule is negatively correlated with metabolic flexibility, independently of the other investigated factors. Due to the statistical correlation found in this study it is not possible to establish or determine if elevated sE-selectin leads to the development of metabolic inflexibility or insulin resistance, or if the development of individual components of MS leads to endothelial dysfunction. The potential mechanism of this process remains unclear. We would like to propose possible explanations of our findings.

First of all, sE-selectin could be only a marker of metabolic inflexibility in obese patients, as well as a marker of endothelial dysfunction. Many data have demonstrated that sE-selectin is a proinflammatory and proatherogenic cytokine which is associated with obesity, insulin resistance, and cardiovascular disease [6, 8, 9, 38]. sE-selectin may represent a link between obesity and related co-morbidities [27], and this cytokine could be an independent predictor of type 2 diabetes [10].

It is widely accepted that impaired endothelium-dependent vasodilatation in the states of insulin resistance results in a decrease in insulin delivery to insulin-sensitive muscle tissue, and decreases glucose uptake in muscles [39, 40]. According to the data, in the insulin-resistant state the overstimulation of mitogen-activated protein kinase (MAPK)-dependent pathways by compensatory hyperinsulinemia in the endothelium resulted in an increased expression of E-selectin [41].

The relationship between sE-selectin and metabolic flexibility has not been studied. We hypothesized that sE-selectin could be involved in the pathogenesis of insulin resistance by modulating the switching between fat and carbohydrate oxidation. We suggest that the theoretical explanation of this finding could be connected with a self-stimulating inflammatory cycle. Many data have highlighted this cycle in the metabolic inflexibility state [4]. It starts from ectopic fat depots in the skeletal muscle, the liver, or the pancreas. These depots may be the source of oxidative stress [21, 22]. ROS (reactive oxygen species), elevated free fatty acids, and overproduction of different cytokines, i.e., interleukin 1*β*, interleukin 6, or tumor necrosis factor* α*, could increase E-selectin [42]. Moreover, it could probably lead to suppressing mitochondrial function, especially the capacity for fuel usage [23].

We proposed the hypothesis that sE-selectin in the insulin resistance state could be responsible for the production of ROS in subjects’ mitochondria. This could lead to respiratory chain dysfunction, and afterward decreased fatty acid beta-oxidation. The consequence of this is metabolic inflexibility. This disturbance could be observed in MS as a result of environmental, genetic, or psychosocial factors interacting through complex networks.

It is widely known that low levels of physical activity together with an over-abundance of food is the main cause of MS, which is also called a “metabolically inflexible phenotype” [43]. Hypercaloric nutrition leads to insulin resistance and oxidative stress due to mitochondrial overload and metabolic inflexibility [4]. The first step in the development of MS is inflammation-induced endothelial dysfunction. This could be responsible for suppressing mitochondrial function and its capacity for fuel usage [23]. As a result of our findings, we would like to suggest that sE-selectin may be associated with reduced mitochondrial function, because it appears that people with MS may exhibit reduced mitochondrial function [11].

The incidence and severity of MS is inversely related to the level of fitness, and is a marker of a sedentary lifestyle [3]. One of the methods to show the severity of MS is the metabolic syndrome *Z*-score. A lower MS *Z*-score means a better, and a higher score indicates a worse profile of individuals [3]. In our study, in the obese group the mean MS *Z*-score was 1.8, and correlated positively with sE-selectin level and negatively with metabolic flexibility and insulin sensitivity. This suggests that a sedentary lifestyle and overeating could be connected with elevated sE-selectin and loss of metabolic flexibility. In the lean group metabolic flexibility was preserved, with lower sE-selectin level and lower MS *Z*-scores.

It should be pointed out that it is still unclear whether insulin resistance precedes metabolic inflexibility or vice versa. Many studies have shown disturbances in fuel shift in insulin-resistant individuals, but the underlying mechanism remains unclear. Insulin resistance may be related to metabolic inflexibility [2]. Metabolic flexibility and insulin sensitivity are improved after weight loss [44]. It is not clear which is first: metabolic inflexibility or insulin resistance. Impaired stimulation of carbohydrate oxidation and impaired inhibition of lipid oxidation in response to insulin could precede peripheral insulin resistance [45]. In this state a decrease in lipid oxidation, and next an increase in lipid accumulation, which is responsible for insulin resistance, can be observed [46]. Interestingly, metabolic flexibility was reduced in individuals with pre-diabetes [47], independent of insulin resistance and obesity [45]. We can speculate that disturbances in fuel selection could be primarily dependent on endothelial dysfunction, i.e., elevated sE-selectin. This cytokine could link endothelial dysfunction and metabolic inflexibility, whereas the development of whole-body insulin resistance could be the next step in the pathogenesis of type 2 diabetes. However, this hypothesis remains unproven and requires further investigation.

In the present study we performed indirect calorimetry, which showed whole-body glucose and lipid oxidation. Non-oxidative glucose metabolism was also estimated by subtracting the glucose oxidation rate during hyperinsulinemia from the whole-body glucose disposal rate. It is impossible to show the tissue-specific substrate metabolism using this method. In humans most glycogen is stored in skeletal muscle (about 500 g), which plays a principal role in glucose homeostasis, whereas the liver stores about 100 g of glycogen. During the hyperinsulinemic euglycemic clamp almost 70–90 % of glucose disposal will be stored as muscle glycogen in healthy subjects [48, 49]. Insulin-mediated non-oxidative glucose metabolism is more or less identical to glycogen synthesis in the skeletal muscle [52, 34]; only about 10 % of the intravenous glucose load is retained by the liver as glycogen [50]. In our study we showed that sE-selectin is also connected with non-oxidative glucose metabolism. The disturbances in NOGM, which reflect the rate of glycogen synthesis, mainly in the skeletal muscle, were observed in the insulin-resistant subjects [41, 51]. This could suggest that elevated sE-selectin level could lead to the development of insulin resistance through decreased glycogen storage, especially in the skeletal muscle.

Another marker of endothelial dysfunction is sICAM-1 [52]. In our study we did not find elevated levels of sICAM-1 in the obese group, or any statistical correlation between sICAM and other assessed parameters. These results are in line with findings by other researchers [11]. According to certain data only sE-selectin is related to insulin resistance, whereas sICAM is associated with hyperglycemia rather than hyperinsulinemia or insulin resistance [53]. In contrast to E-selectin, which is exclusively expressed on endothelial cells, ICAM-1 can also be expressed on leukocytes, fibroblasts, or epithelial cells in different diseases [54]. We investigated relatively young groups without any disease, and we can speculate that sE-selectin could be overproduced as the first molecule in the insulin-resistant state. However, further studies are necessary to confirm this theory. Moreover, the impact of obesity may be stronger on sE-selectin than other endothelial factors.

Adiponectin could have multiple effects on glucose and lipid metabolism [28–30]. An in vitro study showed that adiponectin inhibits the expression of E-selectin [55]. Del Turco et al. [26] demonstrated that adiponectin has a protective role in endothelial dysfunction induced by advanced glycation end products (AGEs), especially by decreasing E-selectin endothelial expression. However, as mentioned above, in multiple regression analysis we demonstrated that the relationship between metabolic flexibility and sE-selectin was independent not only of adiponectin but also of age, BMI, waist circumference, fasting glucose and insulin level, insulin sensitivity, and triglycerides.

We performed the euglycemic hyperinsulinemic clamp to assess insulin sensitivity. This method can be used for accurate evaluation of the rate of whole-body glucose disposal during steady-state hyperinsulinemia. In the last 40 min of the clamp the hyperinsulinemia suppressed the secretion of insulin by the pancreas, as well as lipolysis, and decreased hepatic glucose production to 10–15 % [56]. In cases when hepatic glucose production is insufficiently suppressed by hyperinsulinemia, the *M*_ffm_ value could be underestimated, especially in overweight/obese subjects. The amount of glucose production by the liver could be evaluated using additional techniques, i.e., a glucose tracer, although this was not performed in our study. However, in our research plasma insulin level was maintained beyond 75 mIU L^−1^, and this should be sufficient to suppress most of the hepatic glucose production in the subjects without extreme insulin resistance.

Our study is possibly limited by the fact that we examined only women, and our findings cannot reveal the cause–effect relationship between the estimated parameters.

## Conclusions

Our data suggest that endothelial dysfunction, as assessed by plasma sE-selectin, is inversely, and independent of other estimated factors, associated with metabolic flexibility. An increased serum sE-selectin level in obesity could play an important role in disturbances in carbohydrate and lipid metabolism.
